# EBV latent membrane protein 1 abundance correlates with patient age but not with metastatic behavior in north African nasopharyngeal carcinomas

**DOI:** 10.1186/1743-422X-2-39

**Published:** 2005-04-20

**Authors:** Abdelmajid Khabir, Hela Karray, Sandrine Rodriguez, Mathieu Rosé, Jamel Daoud, Mounir Frikha, Tahia Boudawara, Jaap Middeldorp, Rachid Jlidi, Pierre Busson

**Affiliations:** 1 Laboratoire d'Anatomie et de Cytologie Pathologiques, Hôpital Universitaire Habib Bourguiba, 3029 Sfax, Tunisia; 2 Laboratoire de Bactériologie-Virologie, Hôpital Universitaire Habib Bourguiba, 3029 Sfax, Tunisia; 3 UMR 8126 CNRS/IGR, Institut Gustave Roussy, 94805 Villejuif Cedex, France; 4 Département de Santé Publique, Institut Gustave Roussy, 94805 Villejuif Cedex, France; 5 Service de Radiothérapie, Hôpital Universitaire Habib Bourguiba, 3029 Sfax, Tunisia; 6 Service de Chimiothérapie, Hôpital Universitaire Habib Bourguiba, 3029 Sfax, Tunisia; 7 Dept of Pathology, Free University Hospital, De Boelelaan 1117, 1081 HV Amsterdam, The Netherlands; 8 Laboratoire Privé de Pathologie, Cité-Jardin, 3029 Sfax, Tunisia

## Abstract

**Background:**

Undifferentiated nasopharyngeal carcinomas are rare in a majority of countries but they occur at a high incidence in South China and to a lesser extent in North Africa. They are constantly associated with the Epstein-Barr virus (EBV) regardless of patient geographic origin. In North Africa, the distribution of NPC cases according to patient age is bi-modal with a large group of patients being around 50 years old (80%) and a smaller group below 25 years old. We and others have previously shown that the juvenile form of NPC has distinct biological characteristics including a low amount of p53 and Bcl2 in the tumor tissue and a low level of anti-EBV IgG and IgA in the peripheral blood.

**Results:**

To get more insight on potential oncogenic mechanisms specific of these two forms, LMP1 abundance was assessed in 82 NPC patients of both groups, using immuno-histochemistry and semi-quantitative evaluation of tissue staining. Serum levels of anti-EBV antibodies were simultaneously assessed. For LMP1 staining, we used the S12 antibody which has proven to be more sensitive than the common anti-LMP1 CS1-4 for analysis of tissue sections. In all NPC biopsies, at least a small fraction of cells was positively stained by S12. LMP1 abundance was strongly correlated to patient age, with higher amounts of the viral protein detected in specimens of the juvenile form. In contrast, LMP1 abundance was not correlated to the presence of lymph node or visceral metastases, nor to the risk of metastatic recurrence. It was also independent of the level of circulating anti-EBV antibodies.

**Conclusion:**

The high amount of LMP1 recorded in tumors from young patients confirms that the juvenile form of NPC has specific features regarding not only cellular but also viral gene expression.

## Background

Nasopharyngeal carcinoma has a highly variable incidence depending on the geographic area [1]. It is rare in most countries including Europe and North America [1]. Very high incidence foci are located in South China (as much as 25 per 100,000-year). In addition, there are large areas of intermediate incidence including several countries of North Africa (Tunisia, Algeria and Morocco) and South-East Asia (Vietnam, Indonesia)(between 3 and 8 per 100,000-year). The vast majority of NPCs are undifferentiated (WHO type II and III). They are constantly associated with EBV except for a few cases of differentiated forms (WHO I) occuring in non-endemic areas, often related to tobacco and alcohol consumption [2].

EBV-infection of epithelial cells often results in the production of EBV particles; virus-cell interactions are peculiar in NPC cells where EBV-infection is mainly latent [3]. The full length viral genome is contained in the nuclei of all malignant cells which generally contain several copies of EBV DNA in the form of circular extra-chromosomal elements or episomes. Most viral genes – especially genes involved in the productive viral cycle – are silent, in a very large majority of tumor cells. Only a few viral genes compatible with EBV latency are consistently transcribed in NPC. These genes encode small untranslated RNAs called EBER 1 and 2 (Epstein-Barr encoded RNA) and a nuclear protein called EBNA1 (Epstein-Barr nuclear antigen 1) detected in all NPC biopsies and visualized in the majority of malignant cells. Another EBV protein called LMP1 (Latent membrane protein 1) is frequently detected in NPC biopsies but with wide variations between individual tumors. According to numerous reports from various parts of the world, there are about 50 to 60 % NPC biopsies where LMP1 can be visualized in a majority of malignant cells using conventional immuno-histo-chemistry [4-7]. Recent reports have shown that other EBV proteins – LMP2 and the BARF1 protein – are often expressed in NPC biopsies, probably also with wide quantitative variations but this remains to be substantiated [8,9]. All these viral products EBERs, EBNA1, LMP1, LMP2 and BARF1 (BamH1 A open Reading Frame 1) have oncogenic activity in experimental systems and are suspected to contribute to the malignant phenotype of NPC cells [3,9].

Another aspect of EBV association with NPC is the presence of aberrant levels of circulating antibodies directed against viral proteins, in particular against EBNA1 and lytic cycle antigens, such as EA (early antigen) and VCA (Viral Capsid Antigen) but with low antibody levels against LMP1 [10-13]. Although viral lytic cycle proteins are usually not detected in malignant cells there is a relationship between the tumor mass and the concentration of anti-VCA and EA in the blood. A likely explanation of this paradox could be that a very small fraction of malignant cells entering the lytic productive cycle is sufficient to trigger and sustain antibody response although these cells are not easily detected on tissue sections [14].

While in South China, most NPC patients are between 40 and 60 years old, in North Africa, the distribution of NPC according to age is bi-modal. Beside the main peak of incidence around 50 (80% cases), there is a secondary peak between the age of 10 and 25 (20% cases). Previous reports have shown that the juvenile forms of NPC have some specific clinical features, sometimes reminiscent of malignant lymphomas [15,16]. For example, young NPC patients have a higher rate of lymph node metastases than adult patients and they are subjected to earlier recurrences. On the other hand, there is a good presumption that young NPC patients are cured when the complete remission last more than one year [15]. We and others have previously reported that the juvenile and adult forms of NPC have distinct biological characteristics. P53 and Bcl2 are more abundantly expressed in the adult forms whereas c-kit is more frequently detected in the juvenile form [16-18]. There are also reports showing that anti-VCA and EA antibodies are less abundant in the juvenile form suggesting a lower rate of escape from viral latency in tumors from youg patients [13,19]. LMP1 whose expression is highly variable in NPC specimens is suspected to play a role not only in oncogenesis but also in the maintenance of latency [20]. Therefore the aim of this study was to combine investigations on LMP1 expression with assessment of anti-VCA and EA antibodies in the two age groups of North African NPCs. We have found that LMP1 is expressed at a higher level in the juvenile form of NPC. However there is no direct relationship between LMP1 abundance and a low level of circulating anti-VCA and EA antibodies.

## Results

### Patients and tumor specimens

Primary NPC biopsy samples were collected with informed consent from 82 patients, prior to any treatment, in the Sfax University Hospital, between January 1993 and December 1999. The ages ranged from 10 to 77 years (mean age: 43 years). Twenty two (27%) patients were less than thirty years old. The clinical stage of the disease was determined according to the TNM classification of the AJCC/UICC (1997). Five (6%) patients were at stage II, twenty (25%) patients were at stage III and fifty seven (69%) were at stage IV. NPC histological type was determined on tissue sections according to the World Health Organisation (WHO) classification, resulting in the following distribution : 1/82 keratinising squamous cell carcinoma (SCC, WHO type 1, 1.2%), 52/82 non-keratinizing carcinoma (NKC, WHO type 2, 63%) and 29/82 undifferentiated carcinomas (UC, WHO type 3, 35%). All patients were treated by irradiation of the nasopharynx and/or cervical lymph nodes. Fifty one (62%) were first treated by induction chemotherapy. The follow-up period which was the time between the last day of radiation therapy and either the day of death or the date of the last examination varied from 1 to 116 months.

### LMP1 expression in tumor cells and correlations with clinical data

**Figure 1:**
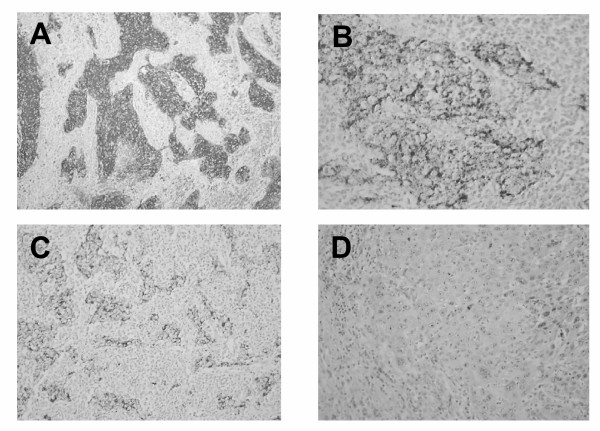
**LMP1 immunostaining on tissue sections of NPC samples. **A. Intense and diffuse LMP1 expression in an NPC biopsy from a 47 year old patient (score 12, 400X) B. Intense LMP1 expression in a limited area in an NPC biopsy from a 17 year old patient (score 7, 600X) C. Moderate and diffuse LMP1 expression in an NPC biopsy from a 44 year patient (score 8, 400X) D. Absence of LMP1 expression in a lung carcinoma biopsy (score 0, 600 X)

Immunohistochemistry using the anti-LMP1 antibody S12 resulted in highly heterogenous staining between tumors from different patients. It was assessed using a scoring system based on the percentage of positive cells and the intensity of staining. Scores of LMP1 varied from 2 to 12 with a mean of 7.6 (+/- 2.6 SD)(Fig. 1 and Table 1). LMP1 staining was also highly heterogeneous within the tumor tissue for each single patient. Both types of heterogeneity did not simply result from the presence of the EBV-negative infiltrating lymphocytes. There were true variations in the amount of LMP1 staining visible in malignant cells, from one patient to another and within a given tumor. We found no NPC specimens with complete absence of S12 staining. Even when staining was minimal, a fraction of cells were nevertheless LMP1-positive with moderate intensity, thus resulting in a score of 2. In contrast, we found a complete absence of staining on sections of lung or laryngeal carcinomas used as negative controls, resulting in a minimal score of 0 (Fig. 1 and data not shown). In the NPC sections with minimal LMP1 staining, we found no specific features of the rare LMP1-positive malignant cells, in terms of cell morphology or relationship with tumor vessels, lymphoid infiltrate or foci of necrosis.

**Table 1:** Variations of the LMP1 score according to clinical and histo-pathological data

		**Number of Specimens**	**Mean Score (SD^a^)**	**p^b^**
Sex				
	Male	57	7.5 (2.6)	p = 0.47
	Female	25	7.9 (2.8)	
Age				
	< 30	22	9.0 (2.4)	p = 0.004
	≥ 30	60	7.1 (2.6)	
Histological type^c^				
	SCC	1	7.1 (3.1)	p = 0.42
	NKC	52	8.0 (2.3)	
	UC	29	7.6 (2.7)	
TNM^d^				
	T2 + T3	40	7.2 (2.6)	p = 0.18
	T4	42	8.0 (2.7)	
	N0	21	7.4 (2.9)	p = 0.67
	N+	61	7.7 (2.6)	
	M0	73	7.8 (2.5)	p = 0.83
	M+	9	7.6 (2.7)	
Metastatic relapse				
	+	24	7.5 (2.8)	p = 0.94
	-	47	7.6 (2.6)	
	NA^e^	11		

^a ^**SD: **standard deviation ^b ^Based on the Student t-test ^c^**Histological type : SCC : **squamous cell carcinoma, **NKC : **non-keratinizing carcinoma, **UC : **undifferentiated carcinoma. ^d ^**Clinical staging: primary tumor extension **classified T2, T3 or T4 according to AJCC/UICC (1997); **regional lymph node extension **classified N0 in the absence of clinical or radiological evidence of lymph node invasion at the initial workup, N+ in the other cases; **metastatic status **defined as M0 in the absence of clinical or radiological evidence of distant metastasis at the initial workup, M+ in the other cases (synchronous metastases). ^e^**NA : **not applicable.

We attempted to find relationships of the LMP1 score with various clinical parameters. We found a highly significant influence of patient age on LMP1 score (p = 0.004)(Table 1). In contrast, we found no relationships with lymph node or extra-nodal metastases at initial examination neither with the occurrence of a metastatic relapse. There was also no relationship with the WHO histological type (Table 1).

### Lack of correlations between LMP1 expression and levels of serum anti-EBV antibodies

As previously reported in other studies, the serum profile of anti-EBV antibodies was not identical in the two age groups of NPC patients [13,21]. Serum levels of anti-VCA and EA IgG were significantly lower in the juvenile form whereas the anti-EA and VCA IgA were undetectable (<10) in majority of young patients (Table 2). Because LMP1 is known to antagonize entry in the lytic cycle in some experimental models we hypothesized that LMP1 might block production of EA and VCA in NPC cells and therefore prevent an increase of circulating antibodies directed to these viral proteins [20]. With this in mind we attempted to find an inverse relationship between the levels of anti-VCA and -EA IgG and IgA on one hand and the level of LMP1 expression in the tumor tissue on the other hand. Using univariate analysis, a significant inverse relationship was found only between the level of LMP1 expression and the level of serum anti-EA IgA (Table 3; p = 0.012). However, this result was not confirmed by multivariate analysis including patient age and title of anti-EA IgA as co-variables. In other words, both LMP1 amounts in the tumor tissue and titles of serum anti-EA IgA are strongly influenced by patient age but there is no direct link between these 2 parameters.

**Table 2:** Variations of anti-VCA and -EA Ig titles according to patient ages

	**Age category **(patient number)		
**EBV-antibody titles**	**< 30 years **(n = 21)	**≥ 30 years **(n = 47)	**p^a^**
**IgG VCA < 320**	**7 **(33,3%)	**4 **(8,5%)	**0,03**
**IgG EA < 40**	**12 **(57,1%)	**6 **(12,8%)	**2.7 × 10^-4^**
**IgA VCA < 10**	**15 **(75%)	**7 **(14,9%)	**< 10^-5^**
**IgA EA < 10**	**16 **(76,2%)	**12 **(25,5%)	**<10^-3^**

^a ^Based on the Fisher exact test

**Table 3:** Variations of the LMP1 score according to serum levels of anti-EBV antibodies

		**Number of Specimens**	**Mean Score (SD^a^)**	**p^b^**
IgA VCA title				
	< 10	22	8.4 (2.9)	p = 0.10
	≥ 10	45	7.2 (2.7)	
	ND^c^	15		
IgA EA title				
	< 10	28	8.6 (2.8)	p = 0.012
	≥ 10	40	6.9 (2.5)	
	ND^c^	14		

^a ^**SD: **standard deviation ^b ^Based on the Student t-test ^c ^**ND : **not determined

## Discussion

Heterogeneity in LMP1 expression in NPC biopsies has been noticed since early studies based on Western blotting. LMP1 amounts can vary from traces only detectable after long exposure of the immunoblots to high levels comparable to those found in EBV-transformed B-lymphocytes [22,23]. For this reason, the rate of NPC specimens recorded as LMP1-postive is highly dependent on the sensitivity of the method used for its detection. For example when using RT-PCR with one round of PCR amplification, LMP1 products are detected in only a fraction of NPC biopsies; in contrast, the percentage of positive samples is often close to 100% when making a second round of PCR using nested primers [24,25]. The same applies to investigations by immuno-histo-chemistry (IHC). According to a recent report by Dietz et al., the percentage of LMP1-postive NPCs markedly increases when using a tyramid-enhancement process instead of conventional tissue staining [26].

In contrast to our study, all previous articles reporting LMP1 detection in NPCs by conventional IHC have recorded a fraction of about 40% specimens as LMP1-negative tumors [4-7]. In most cases, these groups of LMP1-negative tumors were in fact made of 2 categories : specimens with complete absence of LMP1-positive cells and specimens with a percentage of stained cells below an arbitrary threshold of 5 or 10%. In our study, we have found no biopsy completely devoid of LMP1-positive cells. This is probably due to the fact that we have used the S12 antibody which is more sensitive in staining of tissue sections than the CS1-4 antibody from Dako [27]. Hence, to our knowledge, CS1-4 was used in all previous investigations of LMP1 expression in NPC biopsies [4-7]. In addition, we have chosen not to consider any threshold of minimal LMP1 expression; LMP1 staining has been scored even when the protein was visible in a very small fraction of malignant NPC cells.

A large series of studies performed *in vitro *have produced an impressive amount of data suggesting that LMP1 can induce various phenotypic changes consistent with a metastatic behavior. For example in transfected cells, LMP1 can induce the production of the c-Met receptor and of the metallo-protease MMP9 as well as the down-regulation of the E-cadherin [5,28,29]. In this context, it is surprising to find no relationship between LMP1 score and the presence of lymph node or visceral metastases at initial examination or the risk of metastatic recurrence. In this regard, our data are in contrast with two previous reports showing a relationship between LMP1 expression and the frequency of metastases [5,30]. However more recently, Jeon et al. have found a relationship between LMP1 expression and MMP9 expression but not between LMP1 and the presence of metastases [6]. Investigations of LMP1 expression on novel prospective series of NPC patients using the S12 monoclonal antibody might be useful to solve these discrepancies.

## Conclusion

The most striking finding of this study is the observation of a higher level of LMP1 expression in the juvenile form of NPC. It provides clear evidence that this clinical form has specific biological features not only in terms of cellular gene expression but also in terms of latent viral gene expression. From previous studies it was known that anti-VCA and EA IgG and IgA were at a low level in the juvenile form by contrast with the adult form of NPC [13,19]. This observation was confirmed by our own data. However, we found no direct relationship between LMP1 expression and a low level of anti-VCA and EA IgG and IgA. In futures studies, it will be important to investigate in both age-groups of NPCs the status of other EBV-proteins which are suspected to be expressed in this malignancy with a rather heterogenous pattern, for example LMP2A, LMP2B and the BARF1 protein [8,9]. Another issue will be to investigate the anti-LMP1 immune response in the juvenile form of NPCs for example the status of circulating anti-LMP1 antibodies [11].

## Methods

### Pathological diagnosis and immunohistochemical staining of LMP1

All tumor specimens were fixed in Bouin's fixative (75 % saturated picric acid, 25 % formalin, 5% glacial acetic acid) and paraffin-embedded for ligth microscopy and immunohistochemistry. The diagnosis was based on morphological examination after Hematoxylin and Eosin staining. It was further assisted by immuno-staining of Leucocyte Common Antigen and cytokeratin in 29 cases, in order to facilitate the differential diagnosis with a malignant lymphoma or a sarcoma. Tumor sections from all 82 NPC patients were stained with the anti-LMP1 S12 monoclonal antibody. In addition, two squamous carcinomas of the larynx and one squamous lung carcinoma were also stained with S12 and used as negative controls. Five μm sections attached on silanized slides were de-waxed in xylene, rehydrated in graded ethanol, covered with 10 mM citrate buffer (pH 6) and heated in a microwave oven for two consecutive 10 minute periods, at 500 W. They were then incubated for 15 to 30 minutes with the purified primary antibody S12 (0.5 to 1 μg/ml)[27,31]. Primary antibody binding was visualized with biotin-labelled secondary antibodies and a streptavidin-peroxidase complexe using di-aminobenzidine as a chromogenic substrate (LSAB system, Dako).

### Scoring method

Immuno-staining was scored on the basis of the approximate percentage of positive tumor cells and the relative immunostaining intensity. Sections from each biopsies were read and scored independently by two pathologists (AK and RJ) who were blinded to the patient clinical data. Five consecutive microscope fields were analyzed. The differences in scores between the two observers were resolved at a conference microscopy (AK, RJ and TB). The following grading system was adopted to score the number of positive tumor cells: 0, none seen in the section; 1, presence of positive cells even rare but not exceeding 25%; 2, 26 to 50% positive cells; 3, 51 to 75%; and 4, 76 to 100%. Immuno-staining intensity was rated as follows: 0, none; 1, weak; 2, moderate; and 3, intense. When the staining intensity was heterogeneous, each component of the tumor were scored independently and the results were summed. For example, when a specimen contained 50% of the tumor cells with moderate intensity (2 × 2 = 4), 25% of tumor cells with intense immunostaining (1 × 3 = 3), and 25% of cells with weak intensity (1 × 1 = 1), the score was 4 +3 +1 = 8. The maximal possible score was twelve.

### Serological analysis

Serum samples were collected from 68 out of the 82 patients at initial diagnosis. IgG and IgA antibodies to EBV EA and VCA were titrated by indirect immunofluorescence on Raji and P3HR1 cells, respectively [13,32].

### Statistical analysis

LMP1 immunostaining scoring results were expressed as means (standard deviation, SD) and compared using the Student t-test. Variations of anti-EBV antibody titles according to patient age were assessed using the Fisher exact test. To assess relationships between LMP1 score, age and anti-EBV antibody titles, multivariate analysis was carried out using a linear multiple regression (Sas software, version 8, SAS Institute Inc, Cary, NC, USA). All tests were bilateral with a 5% level.

## Competing interests

The author(s) declare that they have no competing interests.

## Authors' contributions

AK, RJ and TB made pathological diagnosis, immunohistochemistry and scoring of immunostaining, HK carried out assessment of serum EBV-antibodies, PB and SR participated in the design and coordination of the study and helped to draft the manuscript, MR performed the statistical analysis, JD and MF gathered clinical data, JM purified the S12 antibody and set up conditions for its use in immunohistochemistry. All authors read and approved the final manuscript.
